# Early clinical and laboratory risk factors of intensive care unit requirement during 2004–2008 dengue epidemics in Singapore: a matched case–control study

**DOI:** 10.1186/s12879-014-0649-2

**Published:** 2014-12-05

**Authors:** Junxiong Pang, Tun-Linn Thein, Yee-Sin Leo, David C Lye

**Affiliations:** Saw Swee Hock School of Public Health, National University of Singapore, Singapore, Singapore; Infectious Diseases, Genome Institute of Singapore, Singapore, Singapore; Communicable Disease Center, Institute of Infectious Diseases and Epidemiology, Tan Tock Seng Hospital, Singapore, Singapore; Department of Medicine, Yong Loo Lin School of Medicine, National University of Singapore, Singapore, Singapore; Lee Kong Chian School of Medicine, Nanyang Technological University, Singapore, Singapore

**Keywords:** Dengue, Intensive care unit, Early risk factors, Matched case–control

## Abstract

**Background:**

Dengue infection can result in severe clinical manifestations requiring intensive care. Effective triage is critical for early clinical management to reduce morbidity and mortality. However, there is limited knowledge on early risk factors of intensive care unit (ICU) requirement. This study aims to identify early clinical and laboratory risk factors of ICU requirement at first presentation in hospital and 24 hours prior to ICU requirement.

**Method:**

A retrospective 1:4 matched case–control study was performed with 27 dengue patients who required ICU, and 108 dengue patients who did not require ICU from year 2004–2008, matched by year of dengue presentation. Univariate and multivariate conditional logistic regression were performed. Optimal predictive models were generated with statistically significant risk factors identified using stepwise forward and backward elimination method.

**Results:**

ICU dengue patients were significantly older (*P*=0.003) and had diabetes (*P*=0.031), compared with non-ICU dengue patients. There were seven deaths among ICU patients at median seven days post fever. At first presentation, the WHO 2009 classification of dengue severity was significantly associated (*P*<0.001) with ICU, but not the WHO 1997 classification. Early clinical risk factors at presentation associated with ICU requirement were hematocrit change ≥20% concurrent with platelet <50 K [95% confidence-interval (CI)=2.46-30.53], hypoproteinemia (95% CI=1.09-19.74), hypotension (95% CI=1.83-31.79) and severe organ involvement (95% CI=3.30-331). Early laboratory risk factors at presentation were neutrophil proportion (95% CI=1.04-1.17), serum urea (95% CI=1.02-1.56) and alanine aminotransferase level (95% CI=1.001-1.06). This predictive model has sensitivity and specificity up to 88%. Early laboratory risk factors at 24 hours prior to ICU were lymphocyte (95% CI=1.03-1.38) and monocyte proportions (95% CI=1.02-1.78), pulse rate (95% CI=1.002-1.14) and blood pressure (95% CI=0.92-0.996). This predictive model has sensitivity and specificity up to 88.9% and 78%, respectively.

**Conclusions:**

This is the first matched case–control study, to our best knowledge, that identified early clinical and laboratory risk factors of ICU requirement during hospitalization. These factors suggested differential pathophysiological background of dengue patients as early as first presentation prior to ICU requirement, which may reflect the pathogenesis of dengue severity. These risk models may facilitate clinicians in triage of patients, after validating in larger independent studies.

**Electronic supplementary material:**

The online version of this article (doi:10.1186/s12879-014-0649-2) contains supplementary material, which is available to authorized users.

## Background

Dengue is identified as the most rapidly spreading mosquito-borne viral disease in the world by the World Health Organization (WHO) in 2012 [1]. Approximately 70% of the population at risk is residing in the WHO Southeast Asia and Western Pacific regions, which make up about 75% of the current global dengue disease burden. This has led to the formation of the Asia Pacific Dengue Strategic Plan (2008–2015) and the recent Global Strategy for Prevention and Control (2012–2020) to reduce the dengue burden [1].

Dengue results in a wide spectrum of non-specific clinical manifestations with unpredictable clinical course and outcome. Patients can be clinically classified as ‘dengue fever’ (DF), ‘dengue hemorraghic fever’ (DHF) or ‘dengue shock syndrome’ (DSS) according to the 1997 WHO dengue classification system and it can be largely asymptomatic [2]. Recently, patients can be clinically classified as ‘probable dengue’, ‘dengue with warning signs’ or ‘severe dengue’ according to the 2009 WHO classification system [3]. Although a large proportion of patients recover after a mild self-limiting disease, a small proportion may progress to develop severe dengue clinical manifestations, which require interventions in intensive care unit (ICU). The progression to severe clinical manifestations is usually unpredictable [3]. Without prompt and appropriate therapy, case fatality rate may exceed 20% [3].

There are currently no systematic studies, but only case series reported [4]–[6] focusing on epidemiological, clinical and laboratory risk factors at first presentation in hospital that are predictive of clinical severity, as defined by the requirement for ICU, instead of the WHO classification criteria. Generally, the proportion of hospitalized dengue patients requiring ICU is low (0.1%-9%) among the high number of hospitalized cases reported [4]–[7], including in Singapore [8],[9]. This is likely due to the fact that there is less severe manifestations in adult dengue patients as compared to pediatric dengue patients, whom are at higher risk of ICU [10]. Furthermore, the timely diagnosis and supportive dengue care management in a more established healthcare system, such as in Singapore may have also resulted in only a small proportion of dengue cases who require ICU admission. Therefore, case series studies and classification may not be generalizable to all populations at risk as well as in different clinical setting and healthcare system. Moreover, with the recommendation of close monitoring of patients with warning signs in hospital [3], there is a greater need to identify these high risk dengue patients 24 hours prior to ICU requirement during hospitalization, which is currently lacking. Henceforth, this study aims to identify and evaluate the early clinical and laboratory risk factors of ICU requirement at first presentation in hospital as well as 24 hours prior to ICU requirement.

## Methods

A retrospective matched case–control study was conducted using anonymized data collected from all adult dengue patients admitted into intensive care unit (ICU) from 1 January 2004 to 31 December 2008 to the Department of Infectious Diseases at Tan Tock Seng Hospital (TTSH). This is the largest hospital in Singapore for the treatment of dengue patients where dengue patients were managed using a standardized dengue care path. Hospitalization criteria were defined in previous published study [8]. In Singapore, dengue infections were predominantly due to dengue serotype 1 (detected in 75% to 100% of dengue samples collected each month) during the epidemics in the year 2004 to 2006, and dengue serotype 2 (detected in up to 91% of dengue samples) during the epidemic in the year 2007 and 2008 [11]. Each case who were admitted into ICU was randomly matched to four patients who did not require intensive care by the year of dengue presentation as controls. Dengue-confirmed patients with positive dengue polymerase chain reaction (PCR) assay, and probable dengue patients with positive dengue immunoglobulin-M (IgM) or immunoglobulin-G (IgG) (Dengue Duo IgM & IgG Rapid Strip, Panbio Diagnostic, Queensland, Australia) and fulfilling either the WHO 1997 or 2009 probable dengue criteria, were included in this study.

According to the WHO 1997 criteria [2], dengue fever (DF) was defined as fever with any two of the following manifestations: headache, retro-orbital pain, myalgia, arthralgia, rash, hemorrhagic manifestations, or leucopenia. Dengue hemorrhagic fever (DHF) was diagnosed when all four criteria of fever, hemorrhagic manifestations, thrombocytopenia (<100 × 10^9^/L) and evidence of plasma leakage (hematocrit change ≥20%, hypoproteinemia or clinical fluid accumulation) were present. Dengue shock syndrome was diagnosed with the presence of rapid and weak pulse with narrow pulse pressure <20 mmHg, or hypotension in a patient with DHF. Probable dengue by WHO 2009 criteria [3] was defined as fever and two of the following: nausea or vomiting, rash, aches and pain, leucopenia, and any warning sign. Warning signs were abdominal pain or tenderness, persistent vomiting, clinical fluid accumulation, mucosal bleed, lethargy or restlessness, hepatomegaly, and hematocrit change ≥20% concurrent with platelet <50 K on the same day. The WHO warning sign of rapid change in hematocrit with rapid drop in platelet count was qualitative. Hence, we had explored previously [9],[12] using a different quantitative definition of hematocrit change of ≥20% with concurrent platelet nadir < 50 K, which is more practical to implement in clinical setting, and had been associated with death outcome [12]. Severe dengue cases fulfil at least one of three criteria: (1) severe plasma leakage: associated with shock (tachycardia >100 or narrow pulse pressure <20 or blood pressure <90 mmHg), (2) severe bleeding: gastrointestinal tract bleeding such as hematemesis or melena or bleeding per rectum, pack cell transfusion or blood transfusion requirement or menorrhagia, (3) severe organ impairment: defined as aspartate or alanine transaminase ≥1000 units/l, encephalopathy, myocarditis and acute renal impairment (creatinine × 2 upper limit of normal for age and sex; based on modification of diet in renal disease equation for glomerular filtration rate = 75 ml/min or baseline estimated from minimum creatinine recorded).

The data at first presentation in hospital and 24 hours prior to ICU requirement were obtained from medical records: demographic, epidemiological, co-morbidity, clinical, laboratory, treatment and outcome data. The severity of the patients as evaluated by the WHO 1997 and 2009 classifications was determined at first presentation and during hospitalisation. The duration of progression to ‘DHF’ or ‘severe dengue’ was determined only for patients who were either classified as ‘DF’ or ‘probable dengue with/without warning signs’ at first presentation. The number of days post presentation (DPP) was used to define the period since the first presentation in hospital. The number of days post fever (DPF) was used to define the period since the day of fever onset.

### Statistical methods

Univariate and multivariate conditional logistic regression were performed to assess the association between the variables and dengue severity as defined by ICU requirement. Conditional logistic regression was used to account for the matching factor selected in the analyses [13]. Matching was performed based on the year of presentation to control for potential confounding by the predominant serotype in each year. Confounding effect was further minimized by performing multivariate conditional logistic regression adjusting for significant demographic & co-morbidity factors associated with ICU requirement only. This parsimonious approach would minimize overloading the multivariable model to achieve higher statistical power to detect true association as the sample size available is small [14],[15]. Continuous variables were illustrated in median with the interquartile range (IQR) for reference. The laboratory variables were analyzed in the continuous format to maximize all the data available, and to minimize reporting bias, especially when the variables were categorized into the expected normal or hypothetical range. For analysis of early risk factors 24 hour prior to ICU requirement, four ICU patients were excluded as they were admitted into ICU less than 24 hour post presentation to hospital. The most optimal predictive model was derived using the stepwise forward and backward elimination method, with the top 10 most significantly associated variables based on the *P*-value of the univariate conditional regression analyses. Only variables with *P*-value <0.05 would remain in the optimal model during the model development. Hosmer–Lemeshow Goodness of fit test was used to check if the model fit the observed dataset with *P* >0.05. Likelihood ratio test was used to assess if a model is more efficient than the other similar model with more significant variables. Fisher’s exact test and Wilcoxon rank-sum test were used for categorical and continuous variables, respectively, for the subgroup analysis of death cases in ICU group. All statistical analyses were performed using Stata 10.0 (Stata Corp., College Station, TX, 2005). All tests were conducted at the 5% level of significance, with conditional odds ratio (COR) and/or adjusted COR (ACOR), *P*-value and corresponding 95% CI reported where applicable.

### Ethics statement

This study was approved by Domain Specific Review Board, National Healthcare Group, Singapore (DSRB-E/08/567) with waiver of informed consent as data were analyzed anonymously.

## Results

A total of 8,123 dengue-infected patients were admitted into Tan Tock Seng Hospital during the dengue epidemics between January 2004 and December 2008, with national incidence rate (per 100,000 population) of about 210 in 2004, 320 in 2005, 70 in 2006, 190 in 2007, and 160 in 2008 [16]. Among these admissions, there were a total of 27 dengue-infected cases (0.3%) that required intensive care interventions in the intensive care unit (ICU). A total of 108 dengue-infected matched controls that did not require intensive care interventions were involved. The median age of the cases was 44 years [Interquartile range (IQR): 36–53 years], with 30% female and 78% Chinese. The median age of the controls was 34 years (IQR: 25–44 years), with 36% female and 71% Chinese (Table 1). Among the cases, there were 48% positive for dengue polymerase chain reaction assay (PCR) and 52% were serology positive. Among the controls, there were 29% positive for dengue PCR, and 71% were serology positive. The median days post fever (DPF) at presentation was 3 (IQR: 3–5 days) and 5 days (IQR: 4–5 days) for cases and controls, respectively. Patients requiring ICU were sicker, requiring earlier admission to hospital. However, there was no statistically significant association observed in gender, ethnic groups, IgG status and median DPF at first presentation with ICU requirements.

**Table 1:** **Demographic and co-morbidities of dengue-infected non-ICU (controls) and ICU (cases) patients**

Variables	Den-infected non-ICU controls (n = 108)	%/(IQR)	Den-infected ICU cases (n = 27)	%/(IQR)	COR	P-value	95% CI	ACOR*	P-value	95% CI
**Median age**	**34**	**(25–44)**	**44**	**(36–53)**	**1.07**	**0.003**	**1.02–1.11**	**1.05**	**0.014**	**1.01–1.10**
Age groups										
14-30	36	33	4	15	1					
30-39	32	30	7	26	3.64	0.138	0.66–20.0	2.48	0.244	0.54–11.41
40-49	24	22	7	26	5.9	0.061	0.92–37.9	4.1	0.126	0.67–25.04
**50-59**	**13**	**12**	**6**	**22**	**8.51**	**0.021**	**1.38–52.6**	**6.9**	**0.027**	**1.25–38.23**
**≥60**	**3**	**2.8**	**3**	**11**	**21.98**	**0.012**	**1.97–245.2**	5.49	0.165	0.50–60.54
Gender										
Female	39	36	8	30	0.74	0.52	0.29–1.87	0.59	0.318	0.21–1.65
Ethnic groups										
Chinese	77	71	21	78	1					
Malay	5	4.6	2	7.4	1.44	0.667	0.28–7.49	2.47	0.36	0.36–17.15
Indian	15	14	2	7.4	0.45	0.344	0.09–2.37	0.39	0.315	0.06–2.47
Others	11	10	2	7.4	0.65	0.606	0.13–3.36	0.95	0.952	0.17–5.29
IgG at presentation										
IgG+	42	39	12	44	1.26	0.596	0.53–2.98	1.19	0.708	0.47–3.03
Median DPF at presentation	5	(4–5)	3	(3–5)	0.75	0.059	0.56–1.01	0.78	0.112	0.58–1.06
**Diabetes mellitus**										
**Yes**	**3**	**2.8**	**4**	**15**	**11.57**	**0.031**	**1.25–1077**	5.53	0.142	0.56–54.15
Hypertension										
Yes	10	9.3	5	19	2.22	0.184	0.69–7.17	0.42	0.296	0.08–2.14
Hyperlipidemia										
Yes	7	6.5	4	15	2.57	0.168	0.67–9.83	1	0.998	0.20–4.87
Cardiac disorder										
Yes	2	1.9	3	11	9.83	0.051	0.99–97.23	3.18	0.402	0.21–47.52

DPF- Days Post Fever; COR- Conditional Odds Ratio; IQR- Interquartile range.

*Adjusted Conditional Odds Ratio (ACOR) was obtained from a multivariate conditional logistic regression with test variables being adjusted by age and diabetes mellitus.

Data in bold are risk factors significantly associated with ICU requirement.

Out of these 27 ICU cases, there were about 93% (n = 25) who had severe plasma leakage, with 81% (n = 22) having shock, 37% (n = 10) had severe bleeding, and 56% (n = 16) had severe organ impairment involving renal, liver and central nervous system during hospitalization. Among the seven death cases, 100% had severe plasma leakage, 71% had severe bleeding, and 100% had severe organ involvement. More descriptions can be found in Additional file 1: Table S1 and in a previously published dengue death study [12]. All ICU patients had intravenous (IV) fluid of about 15 liters on average during hospitalization, and about 40% of the ICU patients had also IV fluid before progression to DHF/DSS. On the contrary, 86% of non-ICU patients had IV fluid of about 4.6 liters on average during hospitalization, and only about 8.6% had IV fluid before progression to DHF/DSS. About 63% of the ICU patients had received platelets infusions, while only 9.7% of the non-ICU patients had received platelets infusions. About 22% of the ICU patients had blood transfusion and only about 1% of the non-ICU patients had blood transfusion. About 30% of the ICU patients had required mechanical ventilation, and none required for the non-ICU patients.

### Demographic and co-morbidity risk factors of ICU

Age groups 50–59 years old [conditional odds ratio (COR) = 8.51; 95% confidence interval (CI) = 1.38-52.6] and equal to or greater than 60 years old (COR = 21.98; 95% CI = 1.97-245) had significantly higher risk of ICU requirement compared with age group 14 to 30 years old. However, after adjusting for diabetes mellitus, only age group 50–59 years old was observed to be a statistically significant risk factor (adjusted COR (ACOR) = 6.9; 95% CI = 1.25-38.23) of ICU requirement (Table 1). Among the co-morbidities analyzed, patients with diabetes mellitus was found to have significantly higher risk (COR = 11.57; 95% CI = 1.25-1077) of ICU requirement compared with patients with no diabetes. After adjusting for age, the risk remained high with ACOR of 5.53 (95% CI = 0.56-54.15), but it was statistically not significant, probably due to the lack of statistical power with a small sample size (Table 1). In addition, patients with cardiac disorder were observed to be at higher risk (ACOR = 3.18; 95% CI = 0.21-47.52) compared with patients without. This may also be a potential risk factor that requires further investigation, even though it was statistically not significant in this study (Table 1).

### Dengue severity classification and clinical outcomes

The WHO 1997 dengue severity classification at presentation was not significantly associated with ICU requirement (ACOR = 1.8; 95% CI = 0.54-6.08), with sensitivity of about 19% and specificity of 87%. However, the WHO 2009 dengue severity classification at presentation was significantly associated with ICU requirement (ACOR = 8.79; 95% CI = 2.65-29.16) (Table 2), with sensitivity of 52% and specificity of 94%. Both the WHO dengue severity classifications as final outcome were observed to be significantly associated with ICU requirement (WHO 1997: ACOR = 2.99; 95% CI = 1.15-7.78) (WHO 2009: ACOR = 116.4; 95% CI = 8.79-1541) (Table 2). The WHO 1997 classification system as final outcome achieved sensitivity and specificity of 67% and 78%, respectively. The WHO 2009 classification system achieved sensitivity and specificity of 89% and 90%, respectively.

**Table 2:** **Severity classification and duration of illness of dengue-infected non-ICU (controls) and ICU (cases) patients**

Variables	Den-infected non-ICU controls (n = 108)	%/(IQR)	Den-infected ICU cases (n = 27)	%/(IQR)	COR	P-value	95% CI	ACOR*	P-value	95% CI
WHO 1997 (Presentation)										
DHF/DSS	14	13	5	19	1.5	0.469	0.50–4.4	1.8	0.34	0.54–6.08
**WHO 2009 (Presentation)**										
**Severe dengue**	**7**	**6.5**	**14**	**52**	**10.3**	**<0.001**	**3.70–28.84**	**8.79**	**<0.001**	**2.65–29.16**
**WHO 1997 (Outcome)**										
**DHF/DSS**	**24**	**22**	**18**	**67**	**7.65**	**<0.001**	**2.74–21.35**	**2.99**	**0.025**	**1.15–7.78**
**WHO 2009 (Outcome)**										
**Severe dengue**	**11**	**10**	**24**	**89**	**73.1**	**<0.001**	**9.82–543.96**	**116.4**	**<0.001**	**8.79–1541**
Median DPP to DHF/DSS	3	(2.3–3.8)	4	(3–5)	1.79	0.206	0.73–4.43	3.08	0.677	0.02–608
Median DPP to Severe dengue	2.5	(1.8–3.3)	3	(3–5)						
**Median LOS in hospital**	**4**	**(3–5)**	**8**	**(6–13.5)**	**1.73**	**<0.001**	**1.30–2.32**	**1.76**	**0.001**	**1.28–2.43**
Median DPF on ICU requirement	N.A		6	(4–7.5)						
Median DPP on ICU requirement	N.A		3	(2–4.5)						
Median LOS in ICU	N.A		3	(2–4)						
Death	0	0	7	26						
Median DPF to death	N.A		7	(7–15.5)						
Median DPP to Death	N.A		3	(2.5–7.5)						

DPF- Days Post Fever; DPP- Days Post Presentation; LOS- Length of Stay; COR- Conditional Odds Ratio; IQR- Interquartile range.

*Adjusted Conditional Odds Ratio (ACOR) was obtained from a multivariate conditional logistic regression with test variables being adjusted by age and diabetes mellitus.

Data in bold are risk factors significantly associated with ICU requirement.

The median days of progression to DHF/DSS and severe dengue for cases was 4 (IQR: 3–5 days) and 3 Days Post Presentation (DPP) (IQR: 3–5 days), respectively. For the controls, the median days was 3 (IQR: 2.3-3.8 days) and 2.5 DPP (IQR: 1.8-3.3 days). The median length of stay (LOS) in hospital was 8 (IQR: 6–13.5 days) and 4 days (IQR: 3–5 days) for cases and controls, respectively. The median Days Post Fever (DPF) and DPP to ICU requirement was 6 (IQR: 4–7.5 days) and 3 (IQR: 2–4.5 days) days respectively. The median LOS in ICU for the cases was 3 days (IQR: 2–4 days). Among the cases, there were seven deaths (case fatality rate = 26%), with median DPF of 7 days (IQR: 7–15.5 days) and median DPP of 3 days to death (IQR: 2.5-7.5 days) (Table 2).

### Early ICU risk factors at first presentation in hospital

At first presentation, warning signs from the WHO 2009 classification were not observed to be significantly associated with ICU requirement. In addition, other signs and symptoms such as hemorrhagic manifestation, rash, leucopenia, nausea/vomiting, ache and pains, thrombocytopenia and tardycardia were not observed to be significantly associated with ICU requirement (Additional file 2: Table S2). Hypoproteinemia (ACOR = 4.65; 95% CI = 1.09-19.74), hypotension (ACOR = 7.63; 95% CI = 1.83-31.79), severe organ involvement (ACOR = 33.04; 95% CI = 3.30-331.3) and hematocrit change ≥20% concurrent with platelet <50 K (ACOR = 8.66; 95% CI = 2.46-30.53) were observed to be significantly associated with ICU requirement (Table 3).

**Table 3:** **Significantly associated clinical and laboratory variables at first presentation of dengue-infected ICU (cases) compared to non-ICU (controls) patients**

Variables	Den-infected non-ICU controls (n = 108)	%/(IQR)	Den-infected ICU cases (n = 27)	%/(IQR)	COR	P-value	95% CI	ACOR*	P-value	95% CI
Hypoproteinemia										
Yes	23	21	10	37	2.28	0.085	0.89–5.85	4.65	0.037	1.09–19.74
Hypotension										
Yes	4	3.7	7	26	8.62	0.002	2.21–33.63	7.63	0.005	1.83–31.79
Severe organ involvement										
Yes	1	0.9	10	37	40	<0.001	5.12–312.5	33.04	0.003	3.30–331.3
Hematocrit change ≥20% concurrent with platelet <50 K										
Yes	7	6.5	11	41	9.4	<0.001	2.95–29.83	8.66	0.001	2.46–30.53
Systolic blood pressure	105	(100–114)	110	(91.5–118.5)	0.98	0.216	0.96–1.01	0.97	0.046	0.94–0.99
Pulse rate	90	(80–96)	95.5	(88.5–102)	1.03	0.049	1.00–1.07	1.05	0.012	1.01–1.09
**Proportion neutrophils**	**60.3**	**(46.2–70.4)**	**76.8**	**(70.7–81.5)**	**1.11**	**<0.001**	**1.05–1.18**	**1.11**	**0.001**	**1.04–1.17**
Proportion lymphocytes	30.3	(22–39)	16.5	(9.8–19.9)	0.90	<0.001	0.85–0.95	0.90	0.001	0.85–0.96
Proportion monocytes	12.5	(8.9–17.3)	7.9	(5.1–9.9)	0.84	0.002	0.76–0.94	0.86	0.004	0.77–0.95
Platelet	68	(44–84)	42.5	(20.3–74.8)	0.98	0.01	0.96–0.99	0.98	0.017	0.96–0.99
**Serum urea**	**3.5**	**(2.6–4.6)**	**5.1**	**(3.7–6.8)**	**1.33**	**0.002**	**1.11–1.58**	**1.26**	**0.030**	**1.02–1.56**
Serum creatinine	77	(65–88.3)	102	(82.3–130)	1.03	0.001	1.01–1.04	1.02	0.006	1.01–1.04
Serum AST	114	(70–195)	156.5	(96.5–516)	1.01	0.017	1.00–1.04	1.01	0.019	1.00–1.04
**Serum ALT**	**74**	**(38–121)**	**92**	**(55.8–293.3)**	**1.01**	**0.012**	**1.001–1.06**	**1.01**	**0.017**	**1.001–1.06**
Serum protein	67	(63–72)	66	(60.5–68.5)	0.89	0.016	0.79–0.98	0.88	0.022	0.79–0.98
Serum albumin	37	(35–40)	37	(31–39)	0.8	0.004	0.69–0.93	0.81	0.006	0.70–0.94

DPF- Days Post Fever; DPP- Days Post Presentation; LOS- Length of Stay; COR- Conditional Odds Ratio; IQR- Interquartile range.

*Adjusted Conditional Odds Ratio (ACOR) was obtained from a multivariate conditional logistic regression with test variables being adjusted by age and diabetes mellitus. Data in bold are risk factors involved in the most optimal predictive model of ICU requirement at first presentation.

Pulse rate (ACOR = 1.05; 95% CI = 1.01-1.09), neutrophil proportion (ACOR = 1.11; 95% CI = 1.04-1.17), serum urea (ACOR = 1.26; 95% CI = 1.02-1.56), creatinine (ACOR = 1.02; 95% CI = 1.01-1.04), aspartate (ACOR = 1.01; 95% CI = 1.001-1.06) (AST) and alanine aminotransferases (ALT), (ACOR = 1.01; 95% CI = 1.001-1.06) at first presentation were positively correlated with ICU requirement (Table 3). On the contrary, lymphocyte (ACOR = 0.90; 95% CI = 0.85-0.96) monocyte proportions (ACOR = 0.86; 95% CI = 0.77-0.95), platelet count (ACOR = 0.98; 95% CI = 0.96-0.99), serum protein (ACOR = 0.88; 95% CI = 0.79-0.98) and albumin (ACOR = 0.81; 95% CI = 0.70-0.94) at first presentation were significantly and negatively correlated with ICU requirement (Table 3).

These early risk factors of ICU requirement at first presentation in hospital were used to create a predictive model. The most optimal model derived was a combination of neutrophil proportion, ALT and serum urea level with an area under the curve (AUC) of 0.92 (Hosmer–Lemeshow Goodness of fit test *P* = 0.52), indicating that the model had good discrimination of patients who are more likely to require ICU when screened at first presentation in hospital. A prognostic index (*P*) was created using this model. Using the default cut-off of *P* = 0, the model had sensitivity of 58.8%, specificity of 96.3%, positive predictive value (PPV) of 76.9% and negative predictive value (NPV) of 91.8%. Using a cut-off of *P* = −1.4 (maximizing sensitivity and specificity), the model had sensitivity of 88.2%, specificity of 88.9%, PPV of 62.5% and NPV of 97.3%. The predicted probability of ICU requirement (*p*_*i*_) at presentation was calculated as follows:

$$\mathrm{Ppresentation}=\ln\left(\frac{\mathrm{pi}}{1-\mathrm{pi}}\right)=0.106x1i+0.004x2i+0.326x3i-10.601$$

For each individual i, *x*_1_ to *x*_3_ are indicator variables taking on respective values, where *x*_1_ represents neutrophil proportion (%), *x*_2_ represents ALT (IU/L) and *x*_3_ represents serum urea (mmol/L).

### Early ICU risk factors at 24 hours prior to ICU requirement

Close monitoring of high risk patients in hospital for ICU requirement may be challenging as clinical conditions can evolve unpredictably. We further explored potential risk factors that can provide stratification at 24 hours prior to ICU requirement after their first presentation in hospital. Pulse rate (COR = 1.07; 95% CI = 1.002-1.14), white cell count (COR = 1.59; 95% CI = 1.04-2.44), lymphocyte (COR = 1.19; 95% CI = 1.03-1.38) and monocyte proportions (COR = 1.35; 95% CI = 1.02-1.78) were observed to be significantly higher in ICU dengue patients 24 hour before ICU requirement compared with these at first presentation (Table 4). On the contrary, blood pressure (COR = 0.96; 95% CI = 0.92-0.996) and hematocrit (COR = 0.81; 95% CI = 0.66-0.99) were observed to be significantly lower in ICU dengue patients 24 hour prior to ICU requirement compared with that at the first presentation (Table 4). The most optimal model derived was a combination of the monocyte and lymphocyte proportion, blood pressure and pulse rate, which had an AUC of 0.94 (Hosmer–Lemeshow Goodness of fit test *P* = 0.96), indicating that the model had good predictive potential of a high risk dengue-infected patient who is likely to require ICU in the next 24 hour. In addition, this model is more efficient than the model that is comprised of only pulse rate and blood pressure, which are known critical factors for ICU requirement, and has an AUC of only 0.74. A prognostic index (*P*) was thus, created using the model with monocyte and lymphocyte proportion, blood pressure and pulse rate. Using the default cut-off of *P* = 0, the model had sensitivity of 74.1%, specificity of 88%, PPV of 87% and NPV of 75.9%. Using a cut-off of *P* = −0.4 (maximizing sensitivity and specificity), the model had sensitivity of 88.9%, specificity of 76%, PPV of 80% and NPV of 86.4%. The predicted probability of ICU requirement in the next 24 hour (*p*_i_) was calculated as follows:

$$P24\mathrm{HpriorICU}=\ln\left(\frac{\mathrm{pi}}{1-\mathrm{pi}}\right)=0.333x1i-0.105x2i+0.079x3i+0.188x4i-5.284$$

**Table 4:** **Significantly associated laboratory variables at 24 h prior to ICU requirement compared to first presentation of dengue-infected ICU (cases) patients**

Variables	Den-infected ICU at presentation (n = 23)^	(IQR)	Den-infected ICU at 24 H prior ICU (n = 23)^	(IQR)	COR	P-value	95% CI
**Systolic blood pressure**	**110**	**(91.5–118.5)**	**90**	**(80–101.5)**	**0.96**	**0.03**	**0.92–0.996**
**Pulse rate**	**95.5**	**(88.5–102)**	**101**	**(90.5–117.5)**	**1.07**	**0.044**	**1.002–1.14**
White cell count	3.3	(2.4–4.2)	4.9	(3–7.4)	1.59	0.033	1.04–2.44
**Proportion lymphocytes**	**16.5**	**(9.8–19.9)**	**23.5**	**(18.1–28.6)**	**1.19**	**0.020**	**1.03–1.38**
**Proportion monocytes**	**7.9**	**(5.1–9.9)**	**12.1**	**(9.1–17.9)**	**1.35**	**0.033**	**1.02–1.78**
Serum hematocrit	44.9	(42.8–47.2)	40.2	(33.6–43.6)	0.81	0.037	0.66–0.99

COR- Conditional Odds Ratio; IQR- Interquartile range.

^- Four cases were excluded as they were admitted into ICU less than 24 hours after first presentation at hospital. Data in bold are risk factors involved in the most optimal predictive model of ICU requirement at 24h prior to ICU requirement.

For each individual i, *x*_1_ to *x*_3_ are indicator variables taking on respective values, where represents *x*_1_ monocyte proportion (%), *x*_2_ blood pressure (mHg), *x*_3_ pulse rate (bpm) and *x*_4_ lymphocyte proportion (%).

### Differential clinical and laboratory features of death cases at first presentation among the ICU cases

A subgroup analysis was performed among the ICU cases to explore the significantly different clinical and laboratory features of death cases at first presentation in hospital (Table 5; Additional file 1: Table S1). The median age for death ICU cases (n = 7) and non-death ICU (n = 20) subgroups was 48 (IQR: 27–50.5) and 43 (IQR: 36.8-54.3) years old, respectively. There were about 30% female in both the death and non-death subgroups (*P* = 1; Additional file 1: Table S1). Based on the 2009 WHO classification, 100% and 85% had severe dengue in the death and non-death subgroups, respectively (*P* = 0.545; Additional file 1: Table S1). The median length of stay in ICU was 3 and 3.5 days for the death and non-death subgroups, respectively (*P* = 0.383; Additional file 1: Table S1). The median length of stay in hospital was 4 and 9 days for death and non-death subgroups (P = 0.029). Importantly, the following characteristics at first presentation were observed to be significantly different between the death and non-death ICU subgroups: having any warning signs (*P* = 0.022), monocyte (*P* = 0.035) and eosinophils proportions (*P* = 0.035), serum AST (*P* = 0.049) and ALT (*P* = 0.010), and protein (*P* = 0.039) (Table 5).

**Table 5:** **Significantly different characteristics between deaths and non-deaths at first presentation in the subgroup analysis of ICU dengue-infected patients**

Variables	Den-infected ICU non-death (n = 20)	%/(IQR)	Den-infected ICU death (n = 7)	%/(IQR)	P-value*
Median LOS in hospital	9	(7–17.25)	4	(3.5–8.5)	0.0285
Median DPF to death	0		3	(2.5–7.5)	
Median DPP to death	0		7	(7–15.5)	
Any warning sign					
Yes	9	45	7	100	0.022^
Proportion monocytes	9.1	(6.55–12.55)	5.4	(3.8–7.15)	0.0348
Proportion eosinophils	0.1	(0–0.4)	0.5	(0.4–0.9)	0.0351
Serum AST	11	(10–17)	19	(16–29)	0.0487
Serum ALT	126	(70.25–263.25)	939	(442.75–1577.75)	0.0103
Serum protein	85.5	(39.25–146.25)	491	(159–653.5)	0.0328

^- Fisher’s exact test.

*- Wilcoxon rank-sum test.

IQR- Interquartile range.

## Discussion

Dengue infection results in a wide spectrum of clinical severity, from self-limiting dengue fever to severe dengue. Timely appropriate monitoring and clinical management, mainly with fluid interventions of dengue patients is critical to reduce morbidity and mortality [2],[3]. Some of these patients may also require critical clinical management in intensive care unit (ICU). Healthcare resources such as ICU beds and close monitoring may be limited during a large epidemic, making effective triage of patients who may require ICU requirement more crucial. However, there is currently a lack of systematic study looking at potential early risk factors for ICU requirement. Early clinical and laboratory risk factors during first presentation at hospital or even 24 hours prior to ICU requirement via regular strict monitoring of risk factors, may assist clinicians to triage these high-risk patients.

From this matched case–control study, dengue patients who were either between 50 to 59 years old or with pre-existing diabetes had about five times the risk of ICU requirement, compared with dengue patients who are less than 30 years old or without diabetes, respectively (Table 1). Age group 50 to 59 years old was also implicated in ICU requirement in a case–control study of dengue fatality [17] and the recent multi-center mortality cohort study in Singapore [12]. However, the age risk factor of dengue hemorrhagic fever (DHF) observed in a dengue 2 epidemic is age group of 30–49 years old [18]. This suggests that different severity, either defined as DHF, fatality or the requirement for ICU requirement, may affect different age groups. Furthermore, age group of 60 years old and above was not observed to be a risk factor of ICU requirement previously [19],[20], which supported the observation in this study. On the contrary, it was observed that the mean age of ICU requirement in New Delhi case series was younger (39.6 years) [4], with about only 10% of the ICU dengue patients aged greater than 55 years, compared with the 22% in this study. In addition, in southern Taiwan, dengue patients 65 years old or above were reported to have a higher fatality risk compared with patients less than 65 years of age [20]. Interestingly, there was no significant difference in age between severe cases requiring ICU and benign cases in a dengue serotype 1 epidemic in New-Caledonia (South Pacific), with mean age of 47 years in both groups [5]. These findings highlighted the potential differences in epidemiology of ICU dengue patients in different geographical regions, which would be interesting for further evaluation. In general, the risk of ICU requirement was observed to increase from age 40 years old and above (Table 1). Diabetes was not an independent risk factor of ICU requirement in this study (even after adjusting for DHF; data not shown), which is also similarly reported in dengue mortality studies [12],[17],[21]. However, diabetes was shown to be an independent risk factor of DHF as an outcome [18],[22], and DHF as an outcome was significantly associated with ICU requirement in this study. This may suggest that having diabetes may not predispose a patient to ICU requirement or death directly, but it may increase the risk of DHF, potentially resulting in ICU requirement. However, caution in interpretation remains as the sample size of cases was small in this study. Furthermore, IgG status was not significantly associated with ICU requirement, hence, secondary infection is not likely to confound the associations observed in this study.

Based on the WHO classification at presentation, patients who fulfilled the WHO 2009 severe dengue classification were at a higher risk of ICU requirement, eight times more than patients classified as non-severe, but not for the WHO 1997 DHF/DSS criteria at first presentation (Table 2). This suggests that the WHO 2009 classification for severe dengue is more sensitive in identifying clinically more severe patients than that of the WHO 1997 classification for DHF/DSS at first presentation, which is also reported in other studies [10],[12],[23]. However, both the WHO 1997 and 2009 classification systems only achieved sensitivity of about 19% and 52%, respectively, in differentiating patients who required ICU requirement at first presentation. On the other hand, both classification systems achieved high specificity of 87% and 94%, respectively. In contrast, as final outcome, the WHO 1997 classification system achieved sensitivity and specificity of about 67% and 78% respectively, and the WHO 2009 classification system achieved sensitivity and specificity of about 89% and 90% respectively. This is comparable to the sensitivity and specificity of identifying patients requiring category III care (which includes ICU requirement) for DHF/DSS (39.0% and 75.5%, respectively) and for severe dengue (92.1% and 78.5%, respectively) as described by Narvaez F et al. [10].

Most warning signs and symptoms at first presentation in this study were not observed as risk factors of ICU requirement in this study, except for hypoproteinemia, hypotension, and hematocrit change ≥20% concurrent with platelet <50 K (Table 3). Other studies also reported that most warning signs were not able to identify patients progressing into DHF, severe dengue, category III care or fatality efficiently [9],[10],[20]. However, gastrointestinal symptoms were proposed as an important warning sign for severe dengue and fatality [20],[22], but it was not associated with ICU requirement in this study. Hypoproteinemia was observed in most (>80%) of the fatal cases in other studies [12],[24], and was observed at first presentation of severe patients who required ICU requirement [5]. Similarly, hypotension was observed in 71% of fatal cases [17], and was observed to be significantly more in severe cases who required ICU [5]. Interestingly, having any warning signs at first presentation is also a risk factor for death among ICU cases (Table 5), besides for severe dengue [25].

The following laboratory parameters at presentation may be potential indicators of ICU requirement at first presentation in hospital, and were reported in ICU requirement case series and/or fatality case–control studies: pulse rate [17], neutrophil [5], lymphocytes and monocytes proportions, platelet count [6],[7],[17],[21],[24], serum urea, creatinine [5],[7],[17], AST, ALT [5]–[7],[17],[24], protein [5],[7] and albumin [5],[7],[17],[24]. In our study, a prognostic model using a combination of neutrophil proportion, ALT and serum urea level provided a sensitivity of 88.2%, specificity of 88.9%, PPV of 62.5% and NPV of 97.3%. This is comparable to the models for predicting severity, defined as death using a model comprised of creatinine, free bilirubin, amylase and platelets with PPV 66.7% and NPV 75.5% [5], and the prediction of DHF in adult patients at presentation with median DPF of 5 days using a model comprised of clinical bleeding, serum urea, serum protein, and lymphocyte proportion with sensitivity 83%, specificity 84%, PPV 18%, NPV 99% [26]–[28]. Serum urea level was a common variable in these models, which suggests urea level in serum may be an important risk factor for severity. However, the level of serum urea is influenced by bleeding, renal failure, and changes in hemoconcentration. Hence, further research is still required to elucidate the impact of urea on dengue severity, which was shown previously to increase complement-fixation of dengue antigen [29]. Monocyte proportion, AST, ALT and serum proteins were also observed to be significantly different between the death and non-death among ICU cases. This suggests that these could be important factors involved in the progression of fatality after ICU requirement, and should be considered for close monitoring during hospitalization. On the contrary, only bandemia was highlighted as a significant risk factor at first presentation between non-fatal and fatal patients [21].

Pulse rate, lymphocyte and monocyte proportions, blood pressure, white cell count, and hematocrit were observed as early risk factors at 24 hours prior to ICU requirement, and were reported in ICU requirement case series and/or fatality case–control studies [4],[17],[21]. In the fatality study, leukocytosis and platelet count were also shown to be significantly different between the first presentation and pre-fatal data [21]. Interestingly, lymphocyte and monocyte proportions of ICU dengue patients at 24 hours prior to ICU requirement was significantly greater compared with that at first presentation, but lower compared with non-ICU dengue patients at first presentation. This may suggest the dynamic differences in immune response between dengue patients who required ICU requirement and dengue patients who did not require ICU requirement were already evident even at first presentation, which is about three to five days post fever. In addition, this may reflect the observation of the immune response changes rapidly over time as a patient progresses from first presentation at hospital to 24 hours before ICU requirement in another study [30]. However, further research is required to validate these observation. A prognostic model using a combination of monocytes and lymphocytes proportion, blood pressure and pulse rate provided a sensitivity of 74.1%, specificity of 88%, PPV of 87% and NPV of 75.9%. Some of these significant variables were also part of the model used for predicting pediatric DHF which comprised of white cell count, monocytes proportion, platelet count, and hematocrit with sensitivity of 97% and specificity of 48% [31]. Furthermore, this model is clinically relevant as pulse rate and blood pressure were known critical factors for ICU requirement, and were selected unbiasedly based on its significance by the regression techniques. Furthermore, this model was observed to be more efficient as compared to the predictive model with only pulse rate and blood pressure as significant variables.

There are some limitations in our study that should be taken into consideration when interpreting the results. First, the study had a relatively small number of patients with ICU requirement that may result in low statistical power to detect true association. However, the power was maximized by using four matched controls to each case. Furthermore, it should be noted that Tan Tock Seng Hospital is a major infectious disease center in Singapore, which provided care for about 40% of all reported dengue patients in Singapore. Second, no dengue serotype data were available for individual study subjects. In order to account for the serotype variable, we used the year of dengue presentation as a surrogate for serotype (as describe in Methods) as well as a matching factor for our selection of controls to minimize confounding effect due to the different predominant serotype each year. Third, there were no data on pharmaceutical intervention prior and during hospitalization to account for potential contribution to disease severity due to chronic diseases. However, since both the ICU and non-ICU dengue patients did not have significant differences in pre-existing comorbidities, the bias due to pharmaceutical interventions on severity outcome should be minimal. Fourth, this model is derived from adult dengue patients presenting to hospital at a median of 5 DPF (IQR = 3–5 days). Hence, the model may be less accurate when used for triage of patients outside this period range, or used at the community healthcare facilities, which may have much shorter days post fever. Further validation studies are still required to confirm the validity and reliability of this model. Fifth, three parameters in our probability equation were laboratory test results, which may not be easily available in resource-poor countries. However, it is hopeful that as the awareness of dengue prevention and clinical management is raised with the guidance of the WHO, there will be an increased in public-private partnerships that can help to resolve this challenge gradually. Sixth, the lack of APACHE II or SOFA scores may have further limit the accuracy and performance of these model development. Lastly, these risk factors and models would not be as useful as expected if there is a lack of commitment of all hospital physicians, particularly in emergency units, to ensure close monitoring of patients during hospitalization using the risk factors identified in this study.

## Conclusion

Studies to assess early risk factors for severity as defined by DHF/DSS and severe dengue for effective triage are important. However, dengue severity as defined by the requirement of ICU is also critical, but lacking. This is the first systematic study, to our best knowledge, that highlighted the differential pathophysiological background of dengue patients at presentation and at 24 hours prior to ICU requirement. Demographic and clinical risk factors for triage at first presentation comprised of age group 50 to 59 years old, pre-existing diabetes, the WHO 2009 classification at first presentation, hypoproteinemia, hypotension and hematocrit change ≥20% concurrent with platelet <50 K were proposed. For laboratory risk factors, two optimal models with significantly associated variables were derived and proposed to complement the WHO classification system. An optimal model at first presentation is comprised of neutrophil proportion, ALT and serum urea level, and another optimal model for assessing severity 24 hours prior to ICU requirement is comprised of monocytes and lymphocytes proportions, blood pressure and pulse rate. This would be useful to identify dengue patients who may require ICU requirement early at presentation as well as during hospitalization for closer monitoring and clinical management to reduce morbidity and mortality, when validated in independent larger studies.

## Additional files

## Electronic supplementary material

Additional file 1: Table S1.: Subgroup analysis of characteristics of death and non-death cases among ICU patients at first presentation. This table shows the overall characteristics at first presentation of death cases compared to non-death cases among ICU patients that were insignificantly different. (DOC 122 KB)

Additional file 2: Table S2.: Signs and symptoms at first presentation of dengue-infected non-ICU (controls) and ICU (cases) patients. This table shows the signs and symptoms that were insignificantly associated to ICU at first presentation in hospital. (DOC 56 KB)

## Abbreviations

ICU: Intensive care unit
CI: Confidence interval
WHO: World Health Organization
DF: Dengue fever
DHF: Dengue hemorrhagic fever
DSS: Dengue shock syndrome
TTSH: Tan Tock Seng Hospital
COR: Conditional odds ratio
ACOR: Adjusted conditional odds ratio
ARDS: Acute respiratory disease syndrome
PCR: Polymerase chain reactions
Pctl: Percentiles
AST: Aspartate aminotransferases
ALT: Alanine aminotransferases
PPV: Positive predictive value
NPV: Negative predictive value
