# Supplementary material for: Early clinical and laboratory risk factors of intensive care unit requirement during 2004–2008 dengue epidemics in Singapore: a matched case–control study
Source: BMC Infect Dis. 2014 Dec 5;14:649. doi: 10.1186/s12879-014-0649-2 (PMC4267742; doi:10.1186/s12879-014-0649-2)
Supplement: Supplementary file 1 — Additional file 1: Table S1.: Subgroup analysis of characteristics of death and non-death cases among ICU patients at first presentation. This table shows the overall characteristics at first presentation of death cases compared to non-death cases among ICU patients that were insignificantly different. (DOC 122 KB) [file 12879_2014_649_MOESM1_ESM.doc]

**Additional file 1: Table S1: Characteristics of Deaths and Non-Deaths Cases at First Presentation Among the ICU Dengue-infected patients.**

| **Variables** | **Den-infected ICU**  **Non-Death (n=20)** | **%** | **Den-infected ICU**  **Death (n=7)** | **%** | **p-value^** |
| --- | --- | --- | --- | --- | --- |
| **Median age**  (25%-75%) | 43 (36.8-54.3) |  | 48 (27-50.5) |  | 0.6779* |
| **Age groups** |  |  |  |  |  |
| 14-30 | 2 | 10 | 2 | 28.6 |  |
| 30-39 | 6 | 30 | 1 | 14.3 |  |
| 40-49 | 5 | 25 | 2 | 28.6 |  |
| 50-59 | 5 | 25 | 1 | 14.3 |  |
| ≥60 | 2 | 10 | 1 | 14.3 | 0.773 |
| **Gender** |  |  |  |  |  |
| Male | 14 | 70 | 5 | 71.4 |  |
| Female | 6 | 30 | 2 | 28.6 | 1 |
| **Ethnic groups** |  |  |  |  |  |
| Chinese | 17 | 85 | 4 | 57.1 |  |
| Malay | 1 | 5 | 1 | 14.3 |  |
| Indian | 2 | 10 | 0 | 0 |  |
| Others | 0 | 0 | 2 | 28.6 | 0.105 |
| **Laboratory diagnosis** |  |  |  |  |  |
| Serology+ | 9 | 45 | 5 | 71.4 |  |
| PCR+ | 11 | 55 | 2 | 28.6 | 0.385 |
| **IgG at presentation** |  |  |  |  |  |
| IgG- | 10 | 50 | 5 | 71.4 |  |
| IgG+ | 10 | 50 | 2 | 28.6 | 0.408 |
| **Median DPF**  **at presentation** (25%-75%) | 3 (3-4.25) |  | 4 (3.5-6) |  | 0.1581* |
| **Year of presentation** |  |  |  |  |  |
| 2004-2006 | 17 | 85 | 5 | 71.4 |  |
| 2007 | 3 | 15 | 2 | 28.6 | 0.580 |
| **Diabetes mellitus** |  |  |  |  |  |
| Yes | 3 | 15 | 1 | 14.3 | 1 |
| **Hypertension** |  |  |  |  |  |
| Yes | 4 | 20 | 1 | 14.3 | 1 |
| **Hyperlipidemia** |  |  |  |  |  |
| Yes | 4 | 20 | 0 | 0 | 0.545 |
| **Cardiac disorder** |  |  |  |  |  |
| Yes | 2 | 10 | 1 | 14.3 | 1 |
| **Asthma** |  |  |  |  |  |
| Yes | 0 | 0 | 0 | 0 |  |
| **WHO 1997**  **(Presentation)** |  |  |  |  |  |
| DF | 16 | 80 | 6 | 85.7 |  |
| DHF/DSS | 4 | 20 | 1 | 14.3 | 1 |
| **WHO 2009**  **(Presentation)** |  |  |  |  |  |
| Non-severe dengue | 12 | 60 | 1 | 14.3 |  |
| Severe dengue | 8 | 40 | 6 | 85.7 | 0.077 |
| **WHO 1997**  **(Outcome)** |  |  |  |  |  |
| DF | 10 | 50 | 4 | 57.1 |  |
| DHF/DSS | 10 | 50 | 3 | 42.9 | 1 |
| **WHO 2009**  **(Outcome)** |  |  |  |  |  |
| Non-severe dengue | 3 | 15 | 0 | 0 |  |
| Severe dengue | 17 | 85 | 7 | 100 | 0.545 |
| **Median DPP to DHF/DSS**  (25%-75%) | 4 (3-5) |  | 3 (2.75-5) |  | 0.6378* |
| **Median DPP to severe dengue**  (25%-75%) | 5 (3-6) |  | 1 |  | 0.1049* |
| **Median DPF on ICU admission**  (25%-75%) | 5 (3.75-7.25) |  | 6 (5.5-7) |  | 0.3144* |
| **Median DPP on ICU admission**  (25%-75%) | 3.5 (2-5) |  | 3 (2-4) |  | 0.8655* |
| **Median LOS in ICU**  (25%-75%) | 3 (2-4) |  | 2 (1.5-5) |  | 0.3826* |
| **Median DPF to death**  (25%-75%) | 0 |  | 3 (2.5-7.5) |  |  |
| **Median DPP to death**  (25%-75%) | 0 |  | 7 (7-15.5) |  |  |
| **Hemorraghic manifestation** |  |  |  |  |  |
| Yes | 6 | 30 | 1 | 14.3 | 0.633 |
| **Any Rash** |  |  |  |  |  |
| Yes | 10 | 50 | 2 | 28.6 | 0.408 |
| **Leucopenia** |  |  |  |  |  |
| Yes | 13 | 65 | 3 | 42.9 | 0.391 |
| **Nausea/ vomiting** |  |  |  |  |  |
| Yes | 13 | 65 | 6 | 85.7 | 0.633 |
| **Aches and pains** |  |  |  |  |  |
| Yes | 15 | 75 | 4 | 57.1 | 0.633 |
| **Abdominal pain/tenderness** |  |  |  |  |  |
| Yes | 5 | 25 | 2 | 28.6 | 1 |
| **Persistent vomiting** |  |  |  |  |  |
| Yes | 0 | 0 | 0 | 0 |  |
| **Clinical fluid accumulation** |  |  |  |  |  |
| Yes | 2 | 10 | 1 | 14.3 | 1 |
| **Mucosal bleed** |  |  |  |  |  |
| Yes | 4 | 20 | 1 | 14.3 | 1 |
| **Lethargy** |  |  |  |  |  |
| Yes | 2 | 10 | 3 | 42.9 | 0.091 |
| **Hepatomegaly** |  |  |  |  |  |
| Yes | 0 | 0 | 0 | 0 |  |
| **Hypoproteinemia** |  |  |  |  |  |
| Yes | 6 | 42.9 | 4 | 80 | 0.303 |
| **Thrombocytopenia** |  |  |  |  |  |
| Yes | 18 | 90 | 7 | 100 | 1 |
| **Tachycardia** |  |  |  |  |  |
| Yes | 5 | 26.32 | 4 | 57.14 | 0.188 |
| **Hypotension** |  |  |  |  |  |
| Yes | 5 | 25 | 2 | 28.57 | 1 |
| **Narrow pulse pressure** |  |  |  |  |  |
| Yes | 1 | 5.56 | 0 | 0 | 1 |
| **Respiratory distress** |  |  |  |  |  |
| Yes | 1 | 5 | 0 | 0 | 1 |
| **Gastrointestinal bleeding** |  |  |  |  |  |
| Yes | 0 | 0 | 0 | 0 |  |
| **Severe bleeding** |  |  |  |  |  |
| Yes | 0 | 0 | 1 | 14.29 | 0.259 |
| **Altered level of consciousness** |  |  |  |  |  |
| Yes | 0 | 0 | 0 | 0 |  |
| **Jaundice** |  |  |  |  |  |
| Yes | 0 | 0 | 0 | 0 |  |
| **Hyperbilirubinemia** |  |  |  |  |  |
| Yes | 0 | 0 | 0 | 0 |  |
| **Severe organ involvement** |  |  |  |  |  |
| Yes | 7 | 35 | 3 | 42.86 | 1 |
| **Hematocrit change of ≥20% with concurrent platelet nadir < 50K** |  |  |  |  |  |
| Yes | 6 | 30 | 5 | 71.4 | 0.084 |
| **Temperature** | 38.4 (37.3-39.0) |  | 37.8  (36.5-39.75) |  | 0.7605* |
| (25%-75%) |  |  |  |  |  |
| **Systolic blood pressure**  (25%-75%) | 107 (92.5-118.5) |  | 115 (95-117) |  | 0.6377* |
| **Pulse rate**  (25%-75%) | 91 (89-99.5) |  | 114 (78.5-121) |  | 0.2471* |
| **Respiratory rate**  (25%-75%) | 20 (18-20) |  | 20 (18.5-20) |  | 1* |
| **Oxygen saturation**  (25%-75%) | 96.5 (95-98.3) |  | 97.5 (96.75-98.25) |  | 0.46289* |
| **White cell count**  (25%-75%) | 3.2 (2.35-4.65) |  | 3.9 (3.6-4.4) |  | 0.4345* |
| **Proportion neutrophils/**  **polymorphs**  (25%-75%) | 76.8 (72.7-79.65) |  | 75.7 (63.75-82.9) |  | 0.7071* |
| **Proportion lymphocytes**  (25%-75%) | 15.1 (8.4-20.9) |  | 19 (15.85-19.6) |  | 0.298* |
| **Proportion atypical reactive lymphocytes**  (25%-75%) | 6 (4-11) |  | 15 (15-15) |  | 0.6547* |
| **Serum hematocrit**  (25%-75%) | 44.4 (43.25-47.95) |  | 45.5 (41.3-48.15) |  | 0.7947* |
| **Platelet**  (25%-75%) | 15.2 (14.3-15.95) |  | 15.5 (14-16.15) |  | 0.9769* |
| **Serum urea**  (25%-75%) | 3.9 (3.5-4.075) |  | 4 (3.3-4.4) |  | 0.6756* |
| **Serum creatinine**  (25%-75%) | 4.85 (3.75-7.25) |  | 5.9 (4.225-6.075) |  | 0.6264* |
| **Serum Bilirubin**  (25%-75%) | 103 (97.5-105) |  | 95 (95-97) |  | 0.3036* |
| **Serum albumin**  (25%-75%) | 66 (65-69.25) |  | 61 (59-62) |  | 0.2455* |

^- Fisher’s exact test

*- Wilcoxon rank-sum test
