# Supplementary material for: Early clinical and laboratory risk factors of intensive care unit requirement during 2004–2008 dengue epidemics in Singapore: a matched case–control study
Source: BMC Infect Dis. 2014 Dec 5;14:649. doi: 10.1186/s12879-014-0649-2 (PMC4267742; doi:10.1186/s12879-014-0649-2)
Supplement: Supplementary file 2 — Additional file 2: Table S2.: Signs and symptoms at first presentation of dengue-infected non-ICU (controls) and ICU (cases) patients. This table shows the signs and symptoms that were insignificantly associated to ICU at first presentation in hospital. (DOC 56 KB) [file 12879_2014_649_MOESM2_ESM.doc]

**Additional file 2: Table S2: Signs and symptoms at first presentation of dengue-infected non-ICU (controls) and ICU (cases) patients.**

| **Variables** | **Den-infected Non-ICU Controls (n=108)** | **%** | **Den-infected ICU**  **Cases**  **(n=27)** | **%** | **COR** | **p-value** | **95% CI** | **ACOR*** | **p-value** | **95% CI** |
| --- | --- | --- | --- | --- | --- | --- | --- | --- | --- | --- |
| **Hemorrhagic manifestation** |  |  |  |  |  |  |  |  |  |  |
| Yes | 45 | 42 | 7 | 26 | 0.51 | 0.153 | 0.21-1.28 | 0.56 | 0.256 | 0.21-1.52 |
| **Any Rash** |  |  |  |  |  |  |  |  |  |  |
| Yes | 58 | 54 | 12 | 44 | 0.69 | 0.389 | 0.29-1.61 | 0.83 | 0.702 | 0.32-2.16 |
| **Leucopenia** |  |  |  |  |  |  |  |  |  |  |
| Yes | 67 | 63 | 16 | 59 | 0.88 | 0.768 | 0.39-2.01 | 0.85 | 0.720 | 0.35-2.08 |
| **Nausea/ vomiting** |  |  |  |  |  |  |  |  |  |  |
| Yes | 69 | 64 | 19 | 70 | 1.38 | 0.506 | 0.53-3.60 | 1.48 | 0.454 | 0.53-4.15 |
| **Aches and pains** |  |  |  |  |  |  |  |  |  |  |
| Yes | 80 | 74 | 19 | 70 | 0.82 | 0.683 | 0.30-2.18 | 0.91 | 0.845 | 0.33-2.46 |
| **Any warning sign** |  |  |  |  |  |  |  |  |  |  |
| Yes | 55 | 51 | 16 | 59 | 1.47 | 0.409 | 0.59 -3.69 | 1.56 | 0.382 | 0.57-4.25 |
| **Abdominal pain/tenderness** |  |  |  |  |  |  |  |  |  |  |
| Yes | 25 | 23 | 7 | 26 | 1.15 | 0.767 | 0.45-2.99 | 1.21 | 0.722 | 0.43-3.42 |
| **Persistent vomiting** |  |  |  |  |  |  |  |  |  |  |
| Yes | 0 | 0 | 0 | 0 |  |  |  |  |  |  |
| **Clinical fluid accumulation** |  |  |  |  |  |  |  |  |  |  |
| Yes | 0 | 0 | 3 | 11 |  |  |  |  |  |  |
| **Thrombocytopenia** |  |  |  |  |  |  |  |  |  |  |
| Yes | 89 | 82 | 25 | 93 | 2.74 | 0.198 | 0.59-12.74 | 2 | 0.397 | 0.40-9.98 |
| **Tachycardia** |  |  |  |  |  |  |  |  |  |  |
| Yes | 19 | 18 | 9 | 33 | 2.19 | 0.090 | 0.89-5.43 | 2.63 | 0.087 | 0.87-7.92 |
| **Severe bleeding** |  |  |  |  |  |  |  |  |  |  |
| Yes | 4 | 3.7 | 1 | 3.7 | 1 | 1 | 0.12-8.95 | 1.03 | 0.979 | 0.11-9.70 |
| **Mucosal bleed** |  |  |  |  |  |  |  |  |  |  |
| Yes | 21 | 19 | 5 | 19 | 0.944 | 0.915 | 0.33-2.72 | 1.27 | 0.679 | 0.41-3.87 |
| **Lethargy** |  |  |  |  |  |  |  |  |  |  |
| Yes | 25 | 23 | 5 | 19 | 0.72 | 0.572 | 0.23-2.27 | 0.71 | 0.593 | 0.20-2.50 |
| **Hepatomegaly** |  |  |  |  |  |  |  |  |  |  |
| Yes | 0 | 0 | 0 | 0 |  |  |  |  |  |  |

COR- Conditional Odds Ratio

*Adjusted Conditional Odds Ratio (ACOR) was obtained from a multivariate conditional logistic regression with test variables being adjusted by age and diabetes mellitus.
